# ‘It’s more emotionally based’: Prince Edward Island horse owner perspectives of horse weight management

**DOI:** 10.1017/awf.2024.9

**Published:** 2024-03-11

**Authors:** Megan Ross, Kathryn Proudfoot, Eileen Campbell Nishimura, Emily Morabito, Katrina Merkies, Jean Mitchell, Caroline Ritter

**Affiliations:** 1 Department of Health Management, Atlantic Veterinary College, University of Prince Edward Island, Charlottetown, PE C1A 4P3, Canada; 2 Department of Land and Food Systems, University of British Columbia, Vancouver, BC V6T 1Z4, Canada; 3 Department of Animal Biosciences, University of Guelph, Guelph, ON N1G 2W1, Canada; 4 Campbell Centre for the Study of Animal Welfare, University of Guelph, Guelph, ON N1G 2W1, Canada; 5 Department of Sociology and Anthropology, University of Prince Edward Island, Charlottetown, PE C1A 4P3, Canada

**Keywords:** animal welfare, behaviour change, decision-making, phenomenology, owner perspectives, public perception

## Abstract

Horse obesity is a growing concern that can result in negative welfare. The role horse owners play in horse weight management is not well understood. This study aimed to: (1) explore the attitudes, beliefs, and perceptions of owners with overweight or obese horses regarding their horses’ weight; and (2) understand the motivators and barriers for owners to implement, improve and maintain weight management-related strategies. A semi-structured interview guide based on the Theoretical Domains Framework was developed. Qualitative interviews were conducted with 24 owners in Prince Edward Island, Canada whose horse(s) were previously classified as overweight or obese by a veterinarian. Interviews were analysed using template analysis, organising patterns in the data into a codebook and overarching themes. Owners believed horse weight management was important, however, their perceived complexity of the issue made the implementation of the weight management practices difficult. Owners held conflicting perceptions, viewing overweight horses as well cared for, yet recognised these horses were at increased risk for negative health outcomes. Ultimately, participants felt emotionally torn about compromising their horse’s mental well-being to address weight issues. Owners considered the practicality of weight-management strategies, the strategies’ effectiveness, and whether recommended strategies aligned with their beliefs regarding good horse care practices. Knowledge was embedded into owners’ understanding of horse weight, however, some highlighted that traditional knowledge dominates the equine industry hindering systemic industry change. Increased understanding of the effectiveness and impacts of weight management strategies on horses and fostering a society that recognises and accepts horses within a healthy weight range are warranted.

## Introduction

As the primary decision-makers for horses, owners are at the forefront of horse welfare, including the management of their horses’ weight (Mueller *et al.* 2018; Hötzel *et al.* 2019). The prevalence of overweight/obese horses is relatively high in Canada (28.6%) (Kosolofski *et al.* 2017) as well as abroad, including the US (18.7%) (Thatcher *et al.* 2012), UK (20.6–54.1%) (Wyse *et al.* 2008; Stephenson *et al.* 2011; Robin *et al.* 2015), and Australia (23.1%) (Potter *et al.* 2016). Commonly, owners have considered underfed or malnourished horses a significant welfare issue (McGowan *et al.* 2010) while overlooking the implications of overweight or obese horses (Potter *et al.* 2016; Morrison *et al.* 2017; Furtado *et al.* 2021a). Further, owners tend to be less inclined to make management-related decisions for their overweight horses compared to horses that are underweight (Potter *et al.* 2016; Morrison *et al.* 2017; Furtado *et al.* 2021a), often perceiving overweight horses to be the result of good horse management practices (Furtado *et al.* 2021a; Busechian *et al.* 2022). However, horse obesity can be detrimental to horse welfare, for example, because of its association with painful and potentially life-threatening conditions (Rioja-Lang *et al.* 2020), such as osteoarthritis (Robles *et al.* 2018), laminitis/founder (Milinovich *et al.* 2006; Frank *et al.* 2010) and strangulating lipomas (i.e. benign adipocyte growths which can result in obstruction of the horses intestines) (Garcia-Seco *et al.* 2005; Johnson *et al.* 2009). Moreover, treatments to reverse the negative effects of obesity on horse health and welfare typically involve removing horses from pastures and herd-mates which can result in negative affective states in the isolated horses (Jaqueth *et al.* 2018; Rochais *et al.* 2018; Rioja-Lang *et al.* 2020).

Current strategies to mitigate horse obesity are not always utilised correctly (e.g. owners do not provide enough exercise, forage reduction and use grazing muzzles inappropriately) (Gordon *et al.* 2009; Shepherd *et al.* 2021). Additionally, studies in the UK have highlighted challenges associated with implementing weight management strategies (Morrison *et al.* 2017; Rendle *et al.* 2018; Furtado *et al.* 2021a, 2022b). For instance, despite owners acknowledging their horse’s overweight or obese condition, they encountered difficulties increasing their horse’s exercise regime due to limited time or riding conditions that they perceived as unsafe, e.g. fear of riding (Furtado *et al.* 2021a). Further, strict rules at livery yards (i.e. referred to as boarding facilities in North America), such as not allowing the use of grazing muzzles or feed changes, can create obstacles for owners when attempting to adjust their horse’s management to offset an ‘obesogenic environment’ (i.e. an environment that promotes obesity in their horses) (Furtado *et al.* 2021a, 2022b). Furtado *et al.* (2022b) identified influences on owners that result in obesity for UK leisure horses, including the views owners have towards husbandry and management practices (e.g. keeping horses ‘naturally’) and external pressures (e.g. social norms and managers of boarding facilities).

To develop strategies aimed at improving the welfare of horses whose lives intersect with humans, it is essential to account for the human-horse relationship and the main factors that affect horse owner behaviour (Luna & Tadich 2019). Qualitative research is considered the most “humanistic and person-centered way of uncovering thoughts and actions of human beings” (Renjith *et al.* 2021) and can provide rigorous data about tacit knowledge (i.e. knowledge that is gained through experience). Qualitative methods facilitate active participation from participants to gain in-depth insights into how knowledge is produced and utilised to effectively design support strategies for animal owners (e.g. Christley & Perkins 2010; Smith *et al.* 2022). To date, limited qualitative research exists in Canada related to horse obesity (DuBois *et al.* 2018a,b) and the role Canadian owners play in managing overweight horses. Thus, the objectives of this study were: (1) to explore the attitudes, beliefs, perceptions, and experiences of owners with overweight or obese horses regarding their horses’ weight; and (2) to understand the motivators and barriers for horse owners to implement, improve and maintain weight management-related strategies.

## Materials and methods

### Participant recruitment

This study was approved by the Research Ethics Board of the University of Prince Edward Island (#6009098). Participants were purposively sampled from a previous horse care project (Ross *et al.* 2023; Mills *et al.* unpublished) based on the criteria that they owned a horse that was classified by an equine veterinarian as overweight or obese, i.e. receiving a score of ≥ 7 on the 9-point Henneke Body Condition Scoring system (Henneke *et al.* 1983). For the current study, twenty-five horse owners met the criteria to be included and were sent email invitations to participate. One owner declined due to travelling and, hence, 24 horse owners were included in the sample. Participants were informed of the intentions of the project (i.e. that their interviews would be transcribed and analysed for publication in scientific journals) and that they could withdraw from the interview at any point. Horse owners selected for this study had similar horse management practices (i.e. keeping horses in outdoor, group environments), were predominantly leisure riders keeping their horses at home, women (Table 1), and experienced a similar phenomenon: owning an overweight or obese horse in Prince Edward Island, Canada (Ross *et al.* 2023). This province typically has harsh winters (i.e. cold, snow and wind) and conducive weather for lush green grass the rest of the year.

**Table 1.** Demographic profile of 24 horse owners in Prince Edward Island, Canada, participating in semi-structured interviews regarding horse weight and weight-management strategies

Demographics	Interview Participants
Age range (years)	24–71
Years of experience (years)	10–58
Discipline (n)	
Leisure	13
Competitive Western	3
Competitive English	4
Breeding	2
Standardbred racing	1
Therapy	1
Gender (n)	
Woman	23
Man	1
Horse housing (n)	
Outdoor full-time	22
Indoor part-time	2
Horse housing (n)	
Group	22
Individual	2
Farm Type (n)	
Boarding facility	1
Manages boarding/coaching facility	2
Home	18
Home, boarding/coaching facility	3

### Data collection methods

A semi-structured interview guide was created based on the Theoretical Domains Framework (TDF) (Atkins *et al.* 2017). The TDF is a validated framework that was developed by synthesising 33 existing behavioural theories into 14 domains (Atkins *et al.* 2017). The interview guide (see Appendix A in Supplementary material) addressed the 14 TDF domains to gather a comprehensive understanding of a wide range of influences that horse owners may experience or consider when making weight management-related decisions.

The semi-structured nature of the interviews allowed for comprehensive responses and an open conversation (Baumbusch 2010). All interviews were conducted on-farm, in-person using a voice recording device (Zoom-H2n, Zoom North America, Hauppauge, NY, USA) and lasted 25 to 80 min (mean: 55 min). The first author (MR) conducted all interviews, and each participant was verbally informed of MR’s status as a graduate student. Further, participants received information on the nature of the project emphasising that the interviews aimed to gain insights into the experiences of horse owners when caring for their horse and were not a test of their knowledge. Slight modifications were made to the interview guide throughout the data collection phase based on participant responses to either clarify questions or allow for more in-depth responses.

### Qualitative approach and research paradigm

This study included a heterogeneous sample of horse owners with experience owning at least one overweight or obese horse in PEI (Table 1). Therefore, the qualitative approach phenomenology was deemed best suited for this study with an emphasis on understanding the lived experience of individuals who have faced a similar phenomenon (Creswell & Poth 2018; Smith & Fieldsend 2021). Consistent with phenomenological principles (Creswell & Poth 2018), MR acknowledges the intersection between objective facts and subjective views regarding overweight or obese horses and best horse management practices. Further, this research adopted an epistemological perspective that sees knowledge as iterative and adaptive and is developed socially and experientially. MR used reflexivity and aimed for a temporary suspension (‘bracketing’) of her own world view (e.g. biases, preconceptions and beliefs) to suspend her current understanding of the research topic with the aim to cultivate curiosity during the research process (LeVasseur 2003). However, MR also recognises that a suspension of world views is not entirely possible (LeVasseur 2003) and that transparency regarding the researchers’ background and potential influences on data collection and analysis is therefore crucial (see *Researcher characteristics and reflexivity*).

### Researcher characteristics and reflexivity

MR considers herself an ‘insider’ within the equestrian community as a 26 year old, middle-class, blonde, white, female with over 15 years of experience riding and owning horses. Similar to most individuals in the North American equestrian industry (Canada: Evans [2020]; United States: Mallory [2022]; US Equestrian [2023]), MR recognises her privilege to be an equestrian athlete. Additionally, MR’s experience with managing a horse with Equine Metabolic Syndrome (EMS), as well as her experience interacting with the participants from a previous horse care research (Ross *et al.* 2023) project as a graduate student created an environment of trust and rapport between researcher and participants. Throughout the research process, MR was supported by a team of researchers with backgrounds in animal welfare, human-horse relationships, social science, and qualitative research.

### Data analysis

Interview transcripts were analysed with the software NVivo 12 (version 1.7.1) using template analysis (Brooks *et al.* 2015). Template analysis allows for flexibility in research approaches as well as a structured system for codebook development (Brooks *et al.* 2015). An initial codebook was developed inductively (i.e. not organised based on preconceived theories or ideas) from the first eight interview transcripts whereby notable statements from participants were labelled with codes or meaning units (i.e. words or short phrases) and patterns were identified. Through an iterative process, the codebook was modified while analysing the following 16 transcripts. Codes and themes were discussed repeatedly throughout the process among the research team. The final codebook (see Appendix B in Supplementary material) included overarching themes which aimed to capture broad conceptual ideas from participant responses as they related to the research objectives.

### Strategies to enhance trustworthiness

To ensure the credibility of the research process (Nowell *et al.* 2017), member checking of transcripts (n = 8 participants) and overall results (n = 24) was carried out to confirm that participants felt that their overall experience and conversations were accurately represented in the results. Additionally, multiple researchers were involved during data analysis, discussing the development of the codebook and themes based on participant responses. Participant quotes were included in the manuscript to illuminate their experiences and support the researchers’ conclusions. The transferability of the research (Nowell *et al.* 2017) was facilitated through descriptions of the participants (Table 1) and the study setting to understand the context of participant responses. To enhance dependability (Nowell *et al.* 2017), MR kept a reflexive journal (Corlett & Mavin 2018) and established an audit trail throughout data collection and analysis. Data saturation (i.e. novel insight relating to the overarching research objectives was no longer occurring) was noted in the reflexive journal at interview 11 (Saunders *et al.* 2018). However, additional interviews assisted with substantiating specific topics.

## Results

**Figure 1.**
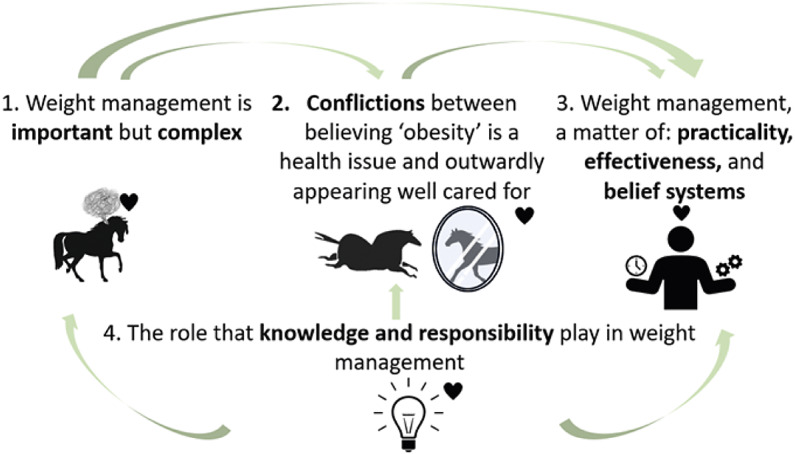
The relationships between the four themes representing the perspectives of the 24 participating horse owners. Themes represent owner attitudes, beliefs and perceptions of horse weight and horse weight management strategies. The small hearts represent owners’ emotions that were embedded into the themes and affected their general understanding and decision-making regarding their horses’ weight.

Four themes were identified to provide a comprehensive understanding of horse owner responses. Figure 1 displays the overarching themes and their inter-relatedness to each other. Ultimately, horse owners believed horse weight management to be important, however, their perceived complexity of the issue along with their conflicting beliefs about overweight or obese horses made the implementation of weight management practices difficult. Owners held conflicting perceptions, viewing overweight horses as well cared for, yet recognised they were at increased risk for negative health outcomes. When owners believed their horses’ weight may be a health risk, they considered the practicality of weight management strategies, their perceived effectiveness of the strategies, and whether the strategies aligned with their beliefs regarding good horse care practices. The knowledge that owners had was embedded into their attitudes, beliefs and perceptions of horse weight and horse weight management strategies. Horse weight management strategies were characterised as malleable, capable of evolving with novel insights and experiences gained by owners. Throughout the four main themes, owners’ emotions were embedded into their attitudes, beliefs and perceptions, and affected their decision-making about horse weight management strategies.

### Weight management is important but complex

Overall, owners recognised the importance of weight management and the consequences of horse management systems that jeopardise a healthy bodyweight. For example, regarding lush pastures, Participant 2 stated: “…*it’s also not good for his health, because, you know, […], especially with the history of laminitis.*” Participant 17 related a horse’s body condition to horses’ ability to be happy: “…*to me, a happy horse can move freely with no pain, is not… is a good body score like not too skinny, not too fat, right? Not killing them with kindness.*” Hence, participants emphasised that healthy horse weight is necessary for horses to live good quality lives.

However, while acknowledging the importance, participants regarded horse weight management as a complex aspect of horse care that requires consideration of a variety of factors to address unhealthy weight gain whilst maintaining horses’ emotional and physical needs. For example, Participant 13 described the complexity of weight management saying: “…*with managing weight, it’s not just feeding them. It’s also veterinary care. It’s also well-being, it’s can they exercise…you know, it’s the whole package.*”

Owners commonly perceived nutritional adjustments and increased exercise as initial steps in addressing overweight horses. However, it was evident that these seemingly straightforward interventions became challenging when the horse began to exhibit metabolic complications, underscoring the complexity of managing horse weight, especially when the cause is metabolic. For example, Participant 12 explains:

“*I look at weight gain as basically a little bit too much food and…not enough exercise…unless you’re getting into metabolic issues, which is another reason you want to keep their weight down because you don’t want to start messing with their insulin…because then you’re getting into trouble.*”.

Participant 17’s horse was diagnosed with Equine Metabolic Syndrome (EMS) and expressed that: “[…] *dealing with a horse with this is extremely frustrating, because* […] *there’s such a lack of knowledge here* [in PEI] *with it.*” Metabolic issues can complicate weight management strategies creating emotional and physical barriers for owners striving to manage their overweight horse. The complexity of weight management was also highlighted when owners discussed how seasons affected their decisions and interpretation of horse weight management: “*If we’re going into winter, I’d much rather have them have a little bit extra weight…And then you kind of don’t want them to have the extra pudge on going into the summer*…” [Participant 14].

Assessments of horse body condition was regarded as complex, needing to understand horses’ backgrounds and individual differences to determine whether they are healthy. Specifically, owners considered horses’ breed, purpose, age, and their predisposition to weight gain when interpreting and making decisions regarding their horses’ weight. Participant 12, for instance, discussed the variation in weight gain with breed: “…*coming from Arabians and Thoroughbreds…I can’t feed this horse enough to get weight, to like a Quarter Horse now where it’s like, okay, I have an apple and I put on 10 pounds.*”

Due to the complexity of managing their horses’ weight, owners discussed how their interaction with their horses allowed them to understand the specific needs of each horse and evaluate changes in their horses’ weight. Participant 8 saw their: “…*horses everyday… So* [I] *notice if one’s starting to kind of lose weight.*”

Owners believed that their interpretation of horse behaviour allowed them to understand how weight can positively or negatively affect horse well-being. For example, Participant 5 described differences in horse behaviour when they are under- versus overweight:

“…*this is what I always tell people. Oh, lovely horse. Oh, she’s so quiet. […] It’s underweight. I say it will not be the same horse when it’s got full weight on them*…[sic] *because it’s got low energy right now. It’s conserving energy. So, it’s not going to be act, like act up. Because it’s underweight, soon as you get them healthy and fat, then all of a sudden, their energy level goes up, they act you know, they’re like, feeling good. And they may start to buck and like, you know, play a lot more while you’re riding them.*”

In general, owners recognised the importance and consequences of horse weight management, and the valence that they placed on their horses’ emotional state as it relates to weight affected their weight management decisions. However, owners’ emotional connection to their horses and the frustration stemming from challenges in helping their horses lose and maintain weight played a role in shaping their willingness and ability to execute, sustain, or enhance their current weight management strategies.

### Conflictions between believing horse ‘obesity’ is a health issue and outwardly appearing well cared for

Despite their recognition of the importance of weight management, and the belief that underweight horses had fewer health risks, owners faced internal conflicts regarding their horses’ weight. Their perceived judgements from others, and the emotional considerations tied to under- versus overweight horses, made it more desirable to have slightly overweight horses. For example:

“…*It’s more emotionally based* [sic] *because actually, for the health of the horse slightly underweight is better. Like if you’re looking at the science and evolution, all that kind of stuff. The horse is not meant to be a fat animal. Okay. So yes, scientifically. I’m slightly underweight, not extremely underweight, but slightly underweight… I think, is healthier than overweight […] But emotionally, I hate to see a horse walking by where you can count all his ribs. See his backbone, you know? That’s, I can’t. Because they’re not, there’s no need for it. They don’t take that much to take care of*” [Participant 5].

It was apparent owners recognised that obese horses are associated with increased health risks. However, owners commonly used language that implied horses were well-cared for if they were slightly overweight (e.g. “*pleasantly plump*” [Participant 7]) and preferred having overweight horses as it was less emotionally taxing for them compared to horses with visible ribs: “*I would say I’m much more like emotionally invested and scared and emotionally labile when she’s like on or at a lower weight*” [Participant 14].

Additionally, many horse owners expressed that other peoples’ perception made it desirable for them to have slightly overweight horses despite the risk of increased negative health consequences. For example, Participant 15 described:

“[…] *the year that I was crying everyday trying to keep the weight on my mare, I knew she was still healthy. Like she was glossy. She was probably a four* [on the body condition scale], *like a hard four on like, weight wise, but I knew I had to go to an inspection, and I knew there was going to be an audience. And the vets were like, you know, she’s fine. She’s given what she has to her foal. She has lots of energy. She’s bright eyed, like her health is good. She’s just showing ribs and hips and I was like, I know but I want, I don’t want her to look like this*…”

Specifically, the desire to avoid negative attention and judgement from the public posed a strong motivation for horse owners to keep their horses’ overweight: “…*I’d rather my horse a little more fat than should be, than not fat enough. (A) because nobody’s gonna call me and call the SPCA* [Society for the Prevention of Cruelty to Animals]…” [Participant 19].

Owners also discussed how some veterinarians shared similar attitudes towards maintaining overweight horses: “…*even the vets like have said that. They’re like, oh it’s just easier if your horses are a little fat*” [Participant 3].

Horse owners described instances where their emotions reflected the perceived emotional state of their horse, and this could act as motivators or barriers for implementing weight management strategies. Owners’ ability to maintain their horses’ positive mental state was a motivator for them to keep their horses healthy, which was dependent on the owners’ understanding and beliefs about what constitutes a healthy horse. However, owners also discussed that doing the right thing, does not always ‘feel good’: “*So we’ll do the grazing muzzle and that may not feel nice to do but it’s the right thing to do. So that’s always hard to differentiate the two*” [Participant 12]. The empathic tendencies of owners towards their horses contributed to their conflicting perceptions. On one hand, they felt distress when horses were thin, while simultaneously being mindful of the negative health associations linked with overweight horses.

### Weight management, a matter of: Practicality, effectiveness, and belief systems

Although horse weight management was deemed important in terms of the health and mental well-being of horses, the complexity of weight management strategies made their implementation, improvement, and maintenance difficult. While horse owners were receptive to modifying their daily horse care regimen, they considered their ability to implement a strategy, the perceived effectiveness of a strategy, as well as their belief systems regarding horse weight and best horse care practices when making decisions related to their implementation of weight management.

Horse owners typically faced the challenges of balancing life, financial and time constraints while caring for their horses. For example, managers of boarding facilities with many horses described not having the: “….*time to go out and give them X amount of hay for their bodyweight so many times a day, they’re in too big of groups, you can’t dictate how much this horse needs*” [Participant 3].

Group housing of horses was prioritised by owners but also made monitoring individual horses’ food intake challenging. Separating horses from their group was often difficult, as described by Participant 23: “[…] *there’s some of those horses that if you try to separate them to feed them, they don’t take to it kindly. So then instead of eating, they stand there, you know, pacing and whinnying, and kicking the wall.*”

Further, equine facilities that are not set up to manage the individual needs of horses also posed an added strain on horse owners’ ability to manage weight: “…*Just again, because I bought an existing facility. I didn’t lay it out how I would want to…I mean, you could keep them in a stall instead of dry lotting them. But…then you have a stall to clean*” [Participant 3].

Exercise for horses was described as a weight management technique as well as a barrier to weight management depending on the horses’ environment, health, and the owners’ perceived ability to execute the strategy. For example, the feasibility for owners to incorporate regular exercise into their daily lives acted as a barrier to achieving healthy weight in their horse(s). Many owners believed keeping horses outdoors in large fields allowed them to maintain a baseline level of natural fitness: “[…] *they do maintain a level of body condition that ultimately they wouldn’t if they were in a small turnout area and in a stall more of the time*” [Participant 10]. However, other owners described that despite having space, it was not enough to maintain their horses’ weight within a healthy range: “…*they’re up on like 10-acre fields, not 100-acre fields, so they’re not travelling 10 kilometers for water a day. So, they’re not getting like that natural fitness*” [Participant 3].

Owners’ perceived effectiveness of weight management strategies also affected the type of management system they chose to implement or maintain. For example, Participant 17 described how the commonly prescribed: “…*dry lot and the 10 pounds of hay… I did try that, and it worked not at all.*” In other instances, owners had mixed feelings about grazing muzzles as a strategy to reduce food intake:

“*It kind of works. I don’t love it. Because when they can’t get: (A) when they’re muzzled they can’t defend themselves so […] there is increased risk of them getting caught up and then I always worry about like, it was really hot this summer so I didn’t like muzzling them during the day when the temperatures really high because just it restricts airflow and I don’t want them to not drink*” [Participant 3].

Owners who had their horses at home had more autonomy over their horses’ care, thus were in some cases better equipped to manage horses outside of traditional management systems. For example, Participant 17 developed a track system instead of keeping their horse on a dry lot: “…*no grass, basically is what they* [veterinarians] *were trying to say. But that’s okay. Because I translated that into, okay, we’re gonna get a track system in, because, I can’t just lock you up in the square, because you’re just going to stand there. That’s not working.*”

Prescribed management systems for overweight horses did not consistently align with owners’ beliefs about best horse care practices. While reducing the risk of weight-related issues acted as a motivator to manage and prevent overweight horses, owners also felt they had to consider the potential impact of weight management strategies on their horses’ emotional well-being. This commonly made owners feel that they had to decide which aspects of their horses’ welfare to prioritise based on their beliefs regarding their horses’ quality of life. For example, Participant 9 described that they put a muzzle on their horse, prioritising their horse’s physical health over their acute emotional state to enhance their horse’s long-term quality of life: “*He’s moving around more. He’s not happy. He’s adjusted to it […] he’d be more unhappy if he was foundered.*” In contrast, Participant 16 felt that keeping horses on pasture was more important than the potential health risks:

“*I’ll see clients and their horse, let’s say tested for Cushing’s […] they can’t go on grass anymore. […] I’m not your vet. But if I personally had your horse, I would let it go on the grass because it’s 22 it’s moving around a lot more, like I think that’s more important than just having it here. And throwing it one flake of hay. Like just, I just, I feel like there’s a better way to do it.*”

Owners also held a common belief that horses should have continuous access to forage that made methods to restrict feed such as muzzling, stabling, decreased time on pasture, and lack of social contact concerning for owners. In particular, forage restriction’s association with ulcers posed a barrier to reduce horse weight:

“…*it comes down to trade-offs because like, research shows again that after four hours of no hay, that’s when you get into the danger zone of like ulcers […] how do you mitigate that without overfeeding them*…” [Participant 3].

Ultimately, owners’ decisions to implement weight management strategies for their horses were shaped by a combination of practical considerations encompassing their perceived ability to execute the strategy and their perceptions of its effectiveness. Moreover, owners’ emotional connections with their horses underscored the significance of aligning strategies with their beliefs regarding what constitutes good care practices and happy horses.

### The role of knowledge and responsibility in weight management

Owners believed they were the most responsible person for managing their horse’s weight and felt accountable for weight-related health conditions. For example, Participant 18 described: “*The laminitis that was, that was really, I mean, I took that very personally that it was my doing, like my doing and that I wasn’t doing right by her*…” However, owners utilised a vast majority of resources to enhance their current knowledge including veterinarians, members of their horse community and literature, and considered the background and credibility of those providing advice. Especially, in some instances, consultation of equine professionals was perceived as a sharing of responsibilities.

“*I think that I am responsible for maintaining my horse’s weight, that depends on getting the right answer from people that are also responsible. So, it’s a little bit tricky, you know… if I get the vet out to visit the horses, at some point, […] then that responsibility has just shifted to the vet*” [Participant 23].

In some cases, owners adapted novel information to make it practical for implementation:

“…*I took her* [nutrition expert] *course just last spring, and it was really hard for me because she’s very scientific. You know, we need to measure this and measure that and so much of this and so much of that and I’m like, okay, but I know I don’t eat like that. I can go to McDonalds when once, you know, every month or something, and it’s okay. So, you know, it’s finding that middle ground, but it’s having all those components of knowledge that I think really helped*” [Participant 17].

Many owners discussed how they have adapted their horse management strategies as they gain experience and guidance from other horse owners and equine professionals. Additionally, having more experience, professional contacts, and a wide array of knowledge provided owners confidence in their horse weight management decisions.

Having knowledge also allowed owners to feel comfortable identifying and addressing their knowledge gap. For example, Participant 6 stated: “…*I feel confident and then wherever my knowledge gaps or skill gap is that’s where my vet comes in*…” However, despite owner respect for veterinarians, they did not consistently feel veterinarians were able to identify solutions for weight-related issues such as laminitis. For example, Participant 17 felt unsupported by their veterinarian: “*I think veterinary is typically my first go-to but having had such poor luck in the laminitis department. […] veterinary medicine was zero help with laminitis.*”

However, this participant also stated that they felt unsupported due to their belief that veterinarians are unsupported: “…*they’re just overwhelmed. So anyway, yeah, unsupported. But it’s, like, unsupported because they’re unsupported sort of thing*” [Participant 17]. Owners commonly suggest that issues surrounding horse care in Canada originate from a systemic level rather than an individual level. For example, Participant 16 explained:

“…*weight management and EMS* [equine metabolic syndrome] *and stuff* [is] *extremely hard here* [in Canada] *because I feel like nobody wants to deal with it and/or they don’t know but they don’t want to say that. So, they just, their go-to is the dry lot and the 10 pounds of hay […]. So, I find myself more and more like trying to find studies they’ve done in the UK because I find they’re like light years ahead of us.*”.

Communication between horse professionals and owners posed an issue when attempting to either gain or transfer knowledge. Managers at boarding facilities often described difficulties communicating with boarders about their horses’ weight. For example, Participant 3 described their boarders’ reactions when telling them their horse was overweight: “…*I’d be like your horse is fat, they’d be running behind my back, basically saying: ‘don’t let her tell you your horse is fat.*’” In contrast, owners also indicated dissatisfaction when managers were uneducated or unwilling to assist with their horses’ needs. This participant described a common feeling among owners which suggested that poor communication and/or varied beliefs about good horse care can make boarding horses at facilities challenging: “…*if* [my horses] *were at a boarding barn, it can be very difficult […] you can ask the* [boarding manager] *to give them* [horses] *more grain. And* [the boarding manager can say]: *well, I don’t think your horse needs it*” [Participant 13].

Inherent knowledge was believed to dominate the horse industry: “…*people who’ve never owned a horse before or never… they don’t have that inherent knowledge that they’ve just accumulated over time*” [Participant 7].

While horse owners express a willingness to adapt their management practices, they discussed a widespread reluctance within the Canadian horse industry to embrace novel approaches that could enhance weight management strategies. Knowledge that is rooted in tradition and years of experience can make changing the horse industry difficult as Participant 3 believed that: “…*we can always do better as an industry, but it’s really hard to, like, make changes when everybody else is still back in the old days*…” It is essential to acknowledge that horse owners take on a significant responsibility for their horses’ care. This responsibility can be emotionally taxing if they do not feel sufficiently supported by the industry. Therefore, owners are calling for the Canadian horse industry to emphasise the distinct roles played by various actors in horse care. The establishment of support systems will promote the advancement of knowledge and adaptable horse care practices.

## Discussion

This study aimed to explore horse owners’ overall experiences with horse weight and horse weight management strategies as well as identifying motivators and barriers related to their implementation. Despite acknowledging the importance of weight management, owners held contradictory beliefs on the relationship between good care practices and horse obesity. Owners perceived that societal acceptance of overweight horses at times hindered optimal weight management strategies. However, owners described the malleability of their management practices based on their evolving knowledge yet cited a lack of external motivation for industry-wide changes.

It is notable to recognise the predominant female participation in this study and the role that horses play in the human-horse relationship. In contemporary times, there has been a noticeable shift in the equine role, moving away from its patriarchal history characterised by wartime applications and cowboy culture, illustrating a ‘feminisation’ of the equine domain (Birke & Brandt 2009; Mazas *et al.* 2013). This feminisation is in tandem with societal shifts towards increased ethical contemplation regarding the treatment of animals (Fraser *et al.* 1997; Fraser 2008). As such, increased awareness of horse emotions is emerging (Finkel & Danby 2019) and is consistent with our findings on the overlap between owner and horse emotions.

These empathic tendencies can act as barriers to the implementation of weight management strategies that impinge on the horse’s emotional state. While emotions play a necessary role in decision-making (Lerner *et al.* 2015), and can have positive associations with animal welfare, empathic and anthropomorphic tendencies have also been reported to have negative influences on animal welfare (Serpell 2002; Apostol *et al.* 2013). For instance, studies (Kienzle *et al.* 1998; German 2016) that have established connections between obesity and the human-animal bond indicate that owners of obese animals are more likely to anthropomorphise their pets. This intricate relationship can complicate owner decision-making, particularly when owners do not accurately interpret horse’s behaviour or weight. Addressing horse owner emotions and the emotions of their respective horses will be critical for communicating effective weight management practices for horses (Tufton *et al.* 2023).

In this study, owners often felt that they had to choose between their horses’ mental and physical health when making decisions related to weight management strategies. This is consistent with Furtado *et al.* (2022b), reporting that horse owners were less inclined to implement weight management strategies that entailed trade-offs for their horses’ welfare. Healthcare decision-making for horses has been described as a multifaceted process rooted in the human-horse relationship (Smith *et al.* 2022); thus, weight management strategies that align with horse owners’ beliefs and values will likely receive greater use in practice (Furtado *et al.* 2021a, 2022b). Ensuring owners recognise that weight management strategies can be temporary and enhance horses’ long-term physical and mental health will be imperative for their uptake in practice. However, for instances when horses are diagnosed with irreversible metabolic issues, weight management strategies may be necessary for the duration of a horse’s life (Cameron *et al.* 2021). Therefore, developing strategies to manage diagnosed weight-related diseases that align with management practices and encourage positive horse affective states must be developed to ensure their uptake by leisure horse owners.

Horse owners in PEI believed proper weight management was imperative for their horses’ overall health and were aware that equine obesity was associated with various health issues such as laminitis, osteoarthritis, and metabolic diseases (Hitchens *et al.* 2016; Furtado *et al.* 2021a; Busechian *et al.* 2022). Despite this, owners commonly chose to keep their horses over- rather than underweight due to their conflicting perceptions about horse weight. The literature consistently reports that horse owners are not always accurate interpreters of their horses’ body condition, specifically suggesting owners to be worse at identifying over- compared to underweight horses (Wyse *et al.* 2008; Potter *et al.* 2016; Morrison *et al.* 2017; Golding *et al.* 2023). This may be related to previous literature reporting the normalisation of overweight horses (Morrison *et al.* 2017).

In addition, qualitative studies report that owners do not consistently differentiate between horse shape, fat, and muscle, creating additional difficulties when deciphering between a horse that is overweight and a horse that is well-muscled (Furtado *et al.* 2021a). Horse owners in the Republic of Ireland that underwent training to use the Henneke Body Condition Scale did not deem it a useful tool for assessing horses body condition since they did not believe it was applicable across breeds (Golding *et al.* 2023). Incorporating the use of real horse images in body condition scales rather than the commonly used drawn images (Henneke *et al.* 1983) will be an important future direction to address owners’ ability to identify overweight horses. This study did not provide any indication that Canadian horse owners utilise body condition scales or receive accurate and consistent information regarding their horses’ weight and body condition. This lack of clarity may contribute to the discrepancy between owners’ understanding of the consequences related to overweight horses and their actual efforts to maintain their horses within the ideal body condition range. To address this gap, future studies may focus on developing a comprehensive database of real horse images that showcase a variety of horses at different points along body condition scales. Such a database will serve as an opportunity for actors within the horse community (i.e. trainers, farriers, veterinarians, nutritionists, researchers) to reflect on their own perceptions of horse weight and, hence, ensure accurate and consistent knowledge transfer. To ensure applicability and usability of body condition scoring tools to a diverse range of owners and their horses, it will be important to include representations of various breeds.

Owners expressed concerns about facing public judgement, suggesting that public perception favours overweight horses which affected their decisions to maintain horses above a healthy weight range. Restricting the use of overweight horses in the media may shift societal perceptions and foster a supportive environment for owners to prioritise healthy horse body conditions. Similar efforts have been utilised by the British Veterinary Association, regarding unhealthy dog breeds, with their 2018 statement discouraging the use of brachycephalic dogs in the media to reduce unhealthy breeding practices (British Veterinary Association 2018). Morrison *et al.* (2017) highlighted the social acceptability of over- compared to underweight horses, reporting that competition judges were more likely to penalise underweight horses compared to overweight horses. Further, recent publications have reported that when compared to expert assessments, judges at horse show competitions tended to underestimate horses’ weight, perpetuating a tendency for owners to maintain horses above a healthy weight range (Munjizun & Phillips 2021). To combat issues with subjective scoring at horse shows in the UK, veterinary assessments of horse body condition have been integrated into horse shows and competitions aiming to redefine healthy horse body condition and incentivise owners to keep their horses within a healthy weight range (Horse Trust 2022). Ensuring consistent feedback from sources, including equestrian judges and veterinarians regarding horse weight and body condition is an important consideration for knowledge dissemination and translation practices.

Horse owners in this project prioritised keeping horses outdoors and in groups (Ross *et al.* 2023) which is consistent with previous literature (Dawkins 1988; Henderson 2007; Hockenhull & Creighton 2015), as it is imperative for horses’ mental and physical well-being. Outdoor housing can also leave horses at the mercy of the weather. Seasonal weight fluctuations are thought to be a common occurrence in horses’ natural environments (Brinkmann *et al.* 2012; Brabender *et al.* 2016). Hence, weather and seasons played an important role in how owners perceived their horses’ weight. Owners often preferred keeping their horses slightly overweight going into the winter and leaner going into spring. This is consistent with previous reports (Hitchens *et al.* 2016) that observed a higher prevalence of overweight/obese horses in the summer compared to the winter.

Owners are often tasked with determining the efficacy of weight management strategies to justify alterations to their management practices. However, owners in this study, consistent with reports from the UK (Furtado *et al.* 2021a,b; Smith *et al.* 2022), expressed feeling limited by practical considerations when deciding on the implementation of intensive husbandry practices or exercise regimes often required for weight management. Commonly prescribed weight management strategies for horses kept outdoors include grazing muzzles, dry lots, and exercise (Glunk *et al.* 2014). However, these methods were often viewed as impractical or ineffective at combating issues surrounding outdoors environments conducive to weight gain. For example, in line with previous reports (Cameron *et al.* 2021), owners in our study noted that grazing muzzles are not always effective as horses can easily remove them. Additionally, some researchers advise removing the muzzle after 12 h, however, this can induce compensatory eating patterns resulting in horses consuming the same amount of grass they would have done so within 24 h without a grazing muzzle (Longland *et al.* 2016; Davis *et al.* 2020).

Dry lots also did not align with owners’ beliefs about good horse management practices, with them believing that dry lots were not always effective as it decreased horses’ ability to maintain natural fitness and led to boredom. Graham-Thiers and Bowen (2013) reported that large pastures improved a horse’s ability to maintain fitness. However, participants in this study mentioned that despite having space, it was often insufficient to maintain their horse’s weight. British nutritionists and veterinarians discussed that the quality of pastures in the UK were often originally meant for cattle, and hence were too nutritious for horses’ daily requirements (Watts 2004; Furtado *et al.* 2021b). PEI also hosts substantial agricultural activities contributing to high quality grass (Leger *et al.* 2000), posing a risk for horse obesity (i.e. an obesogenic environment), underscoring the challenges with maintaining horses at an ideal weight when kept on pasture (Christie *et al.* 2006). In other parts of the world, track (Hampson *et al.* 2010) and dynamic feeding (Laat *et al.* 2016) systems have been used successfully to manage overweight horses by encouraging movement without the risk of superfluous grass intake (Cameron *et al.* 2021; Furtado *et al.* 2022a). In this study, only one participant reported using a track system. Promoting and raising awareness about alternative restricted grazing methods such as track and dynamic feeding systems as preventative management for horse weight may boost their adoption among Canadian horse owners. However, the cost of resources to care for horses seemed to limit some owners’ ability to adapt their management practices in this study and others (Cameron *et al.* 2021; Furtado *et al.* 2022a) despite external sources reporting socioeconomic information of horse owners as predominately being middle class or above (Evans 2011; DuBois *et al.* 2018b). Addressing the impact of various factors such as cost, owners’ beliefs, education, and behaviours will be important future directions to support PEI horse owners who are less inclined or unable to implement alternative restricted grazing measures.

Owners in this study commonly reported concerns with ulcers when minimising forage intake. However, research indicates that horses kept on pasture with supplementation of vitamins and minerals can maintain their bodyweight during grass-growing seasons without additional forage (Williams *et al.* 2020). Ulcers are commonly linked to stress and intense management systems; therefore, leisure horses living in groups with ample outdoor space have a generally low risk of developing ulcers (Feige *et al.* 2002; Chameroy *et al.* 2006; Ward *et al.* 2015). To the authors’ knowledge, it is unknown if these misconceptions arise from poor knowledge dissemination or from misconceptions by those disseminating knowledge, such as veterinarians. Clarifying the causes of ulcers in horses such as their confounding associations between forage consumption and management presents important avenues for future research (Feige *et al.* 2002; Chameroy *et al.* 2006; Ward *et al.* 2015).

In this study, horse weight management practices were viewed as iterative, or ever-changing based on past experiences and knowledge gained through external resources such as peers in their horse community and professionals such as nutritionists and veterinarians. As such, owners discussed that experience-based and inherent knowledge dominated the horse industry. Since horse owners in PEI commonly rely upon each other as an information source (Ross *et al.* 2023) this may pose issues for horse owners due to inconsistent or inaccurate circulation of information. Communication of information between horse owners and professionals will be an important component in enhancing the transfer of information and practical application. For example, providing barn managers and coaches with resources to support their boarders and riders may enhance communication. Additionally, creating a holistic understanding of horse weight management via systems thinking (Luke *et al.* 2022) will promote communication from collaborators such as nutritionists, alternative medicine professionals, farriers and trainers to create additional avenues for owners to formulate and exchange knowledge. Communicative research methods such as this set the stage for future participatory programmes which have been successfully adopted in other disciplines such as improved veterinary confidence during client communication (Lightfoot *et al.* 2020). This will, in turn, improve the quality of circulating information and alleviate pressure placed on veterinarians as a primary knowledge source.

## Animal welfare implications and conclusion

Horses rely upon their owners to provide them with adequate care, placing owners at the gateway to improved horse welfare. Therefore, working with owners to address the barriers to weight management is imperative to improve horse welfare. To the authors’ knowledge, this study is the first to highlight Canadian horse owner perspectives of their horse weight management strategies. The multifaceted barriers and motivators that influence horse owners in PEI when making weight-related management decisions suggest the need to employ holistic knowledge dissemination strategies that account for humans’ emotional attachments to their horses. Further, the deeply ingrained societal paradigms and owners’ inherent beliefs regarding what constitutes a healthy horse poses significant obstacles to achieving healthy horse weights. Therefore, it is critical to shift the societal paradigms surrounding horse weight and work collaboratively with owners to develop tailored solutions that meet their horses’ specific needs. Strategies include facilitating weight management practices that are practical, effective and align with owners’ beliefs and values, particularly those reluctant to sacrifice their horses’ time outdoors or in groups. Shifting the social norm regarding healthy horse weight can lead to fostering a society that recognises and accepts horses within a healthy weight range. Results from this study suggest the need for collaboration between the Canadian equine research and social science researchers to further understand how various actors within the equestrian industry affect horse weight management, and the role that emotions play in weight-management recommendations and decisions.

## Supporting information

Ross et al. supplementary material 1 Ross et al. supplementary material

Ross et al. supplementary material 2 Ross et al. supplementary material

## Supplementary material

The supplementary material for this article can be found at http://doi.org/10.1017/awf.2024.9.
