# Supplementary material for: ‘It’s more emotionally based’: Prince Edward Island horse owner perspectives of horse weight management
Source: Anim Welf. 2024 Mar 11;33:e14. doi: 10.1017/awf.2024.9 (PMC10951667; doi:10.1017/awf.2024.9)
Supplement: Ross et al. supplementary material 1 — Ross et al. supplementary material [file S0962728624000095sup001.pdf]

‘It’s more emotionally based’: Owner perspectives of horse weight management

Appendix A: Interview Guide Template

Researcher Positionality

I am a MSc Student at the University of Prince Edward Island, and this project aims to understand horse owners' experiences and perceptions related to the care of their horses. I am not only a student, but I also own two horses and have experienced the joys and challenges that arise when caring for them. There are no right or wrong answers to any of the questions I ask. I am excited to hear about your personal experiences, perspectives, and opinions related to the care of your horses.

Do you have any questions before we get started?

**Introduction**
1. In a few sentences, please tell me a bit about your horse and yourself.<br>2. Follow-up: What made you want to be part of the horse community

**Horse Housing-Related Questions**
3. What do you consider important when making decisions related to how you care for your horse?<br>4. What does it mean for a horse to have a good life?<br>*If they talk about any of the following aspects of welfare, then follow-up with the probing questions:*

**Theoretical Domain Framework (TDF):<br>Horse Weight Management**	
**TDF Domain**	**Question**
**Intention**	• How much does managing your horses’ weight affect their ability to live a good life?
**Memory, Attention, and Decisions**	• How often do you consider your horse’s weight throughout your care routine with your horse? And why?
**Beliefs in Capabilities**<br><br>**Skills**<br><br>**Knowledge**	• Describe how difficult or easy is it to manage your horse’s weight?<br>• Do you feel you have sufficient knowledge to effectively manage your horse’s weight?<br>• As a horse owner, do you feel capable of managing your horse’s weight?<br>• Describe the skills you feel you have to manage your horses’ weight?
**Motivation**<br><br>**Goals**	• How much do you want to, or how important is it to you to manage your horse’s weight?<br>• Follow-up if not important: what other horse care factors do think are more important?

	• Can you describe some of the motivating factors that encourage you to manage your horse(s) weight?<br>• What are some of the barriers to managing your horses' weight?<br>• What does maintaining a horse within a healthy weight help you achieve?
**Social Influences**	• To what extent do you consider the opinions of other non-horse-related people when making decisions around your horse's weight management?<br>• How do the individuals within your equestrian community influence the decisions you make related to your horse's weight?
**Behavioural Regulation**<br><br>**Social and Professional Role and Identity**	• What systems do you have in place or tactics that you could use for monitoring whether you are successfully managing your horse's weight?<br>• Follow-up: How confident are you in the management strategies you have employed to help a horse lose or gain weight? (Optimism)<br>• Who do you think is most responsible for managing your horses' weight?<br>• Who do you think is most responsible to help guide your decision-making related to your horses' weight?<br>• Do you think that you are sufficiently supported by government policy and/or Equestrian Organizations to manage your horses' weight?<br>• Follow-up: In what ways do you think you could be better supported?
**Beliefs in Consequences**	• Do you think it is better for a horse to be overweight or underweight? Please describe why you picked one over the other (or neither).<br>• Probe: what do you think are the health effects of under or overweight horses?<br>• How do you know/judge if a horse is at a good weight or within a healthy range?
**Optimism**<br><br>**Emotions**	• Describe how confident are you that maintaining your horse's weight within a 'healthy weight' range will enhance their quality of life?<br>• Describe how confident are you that maintaining a horse outside of a healthy weight range will decrease their quality of life?<br>• How would you generally describe the emotions that arise when you think about managing your horse's weight?
**Environment, Context, and Resources**	• To what extent do you believe the environment in which your horse lives impact your horse's weight?<br>• Follow-up: In what ways do you manage your horse's environment to help with their weight?

**Concluding Question**	• Before moving on to my final question, is there anything else you would like to add, that we have not covered?<br>• How, if at all, has your involvement in this study (including Molly's project) changed your overall perception of horse care?
