# Supplementary material for: ‘It’s more emotionally based’: Prince Edward Island horse owner perspectives of horse weight management
Source: Anim Welf. 2024 Mar 11;33:e14. doi: 10.1017/awf.2024.9 (PMC10951667; doi:10.1017/awf.2024.9)
Supplement: Ross et al. supplementary material 2 — Ross et al. supplementary material [file S0962728624000095sup002.pdf]

“It’s more emotionally based”: owner perspectives of horse weight management

Appendix B: Codebook

Objectives:

1. explore the attitudes, beliefs, and perceptions of owners with overweight horses regarding their horses’ weight and
2. to understand the motivators and barriers for horse owners to implement, improve, or maintain weight management-related strategies

**Theme**	**Parent Code**<br>Parent code description	**Child code**<br>Child code description
**Horse weight management is important but complex**	**Top priority for horse care**<br>• Responses that discuss the importance of weight management (e.g., body condition, fitness, nutrition)	
	**Nutrition is integral to weight management**<br>• Any context when nutrition is discussed in relation to managing their horses’ overall well-being	
	**Relationship between fitness, horse shape, and weight management**<br>• Responses related to fitness as a management technique or barrier to weight management<br>• Statements of how fitness relates to the shape of the horse (e.g., topline) and/or how it plays a role in the way in which owners assess their horses’ body condition	
	**Horse-human interaction**<br>• Responses related to the relationship owners have with their horses daily, allowing them to understand their horse’s unique behaviour to monitor their weight and overall health	
	**Patience is key**<br>• When owners emphasize that weight management “takes time”	
	**Horse-specific weight management**<br>• When owners consider their horses’ individual differences (e.g. age, discipline, breed, personality, and lifestyle) when making decisions related to their horse’s weight	
	**Seasonal-specific weight management**<br>• Responses related to how their management strategies may be altered based on the season and the weather	
	**Recognition that underweight is better from a physical health perspective**<br>• When owners discuss any negative consequence of overweight horses	**Owner perceptions of thin or ‘underweight’ horses**<br>• When owners describe thin or underweight horses
		**Owner perception of overweight horses**<br>• When owners describe overweight horses

**Conflictions between believing 'obesity' is a health issue and outwardly appearing well cared for**	• Responses that directly state that being underweight has fewer health consequences	
	**Food is love**<br>• Responses indicating that feeding horses indicates good standard of care for horses	**Emotionally based to keep horses slightly overweight**<br>• Owners' indication that their emotions play a role in their preference for slightly overweight horses
		**Horses that eat are healthy**<br>• Responses related to horses eating as a measure of their overall health
		**External perceptions**<br>• When owners discuss feeling distressed about others' perception of their horse and its quality of care if their horse is underweight.
**Implementing and improving weight management strategies: a matter of effectiveness, practicality, and belief systems**	**Balancing life and horse care**<br>• Owner responses that discuss how they have to consider aspects of their life outside of horse care when making decisions about managing weight	**Weight management requires more intense management**<br>• Responses related to overweight horses requiring more management
	**Horses' quality of life**<br>• When owners discuss that they make decisions based on their perception of their horses having a good quality of life	**Balancing horses' emotional and physical needs**<br>• Responses related to owners considering trade-offs between different aspects of their horses' health
		**Simulating a natural environment**<br>• Owners discussing weight management in relation to horses "living naturally"
	**Mirroring the horses' emotions**<br>• When owners discuss mirroring the emotional states of their horses and/or how that plays a role in their decisions regarding their horses' weight	
	**Beliefs about horse weight management strategies influence horse owner decision making**<br>• Responses related to owners' beliefs about best horse care practices and their perceived effectiveness of weight management strategies, determines whether they choose to implement or maintain a weight management strategy	
	**Knowledge shapes beliefs and decisions**<br>• When owners discuss that the knowledge they have affects the beliefs they have about weight management strategies	**Adapting management with the evolution of knowledge**<br>• When owners express how their management strategies have changed over time and/or their willingness to learn more and appreciation for alternative perspectives

<table>
<tr>
<td rowspan="6">The role of knowledge and responsibility in horse weight management</td>
<td>Growing knowledge: evidence to practice gap
Responses that suggest it is difficult to add knowledge to the horse industry and that there is a lack of intrinsic and extrinsic motivation to grow owners’ knowledge base in the Canadian horse industry</td>
<td>Inherent knowledge dominates the industry
Responses pertaining to how most horse knowledge is gained through experience and tradition and/or that there is minimal external motivation to adapt knowledge base</td>
</tr>
<tr>
<td>Knowledge is empowering
When owners discuss or suggest that having knowledge allows for ease of decision making</td>
<td>Support network
When owners discuss the relationship between having a network of assistance from experienced horse individuals and their confidence in making decisions</td>
</tr>
<tr>
<td rowspan="4">Synthesis of information to form knowledge and make decisions
When owners discuss utilizing resources and aspects that they take into consideration when deciding what to do with the information</td>
<td>Boarding facilities: help or hindrance?
How boarding facilities affected owners’ opinions, experiences, and knowledge</td>
</tr>
<tr>
<td>Veterinary role in weight management
Owners discuss their perceptions of the veterinarian’s role in horse weight management</td>
</tr>
<tr>
<td>Owners take responsibility for their horses’ weight
When owners discuss their own role in managing their horses’ weight</td>
</tr>
<tr>
<td>Confusion and distrust in nutrition companies
Responses related to owners’ confusion around what to feed their horses and/or that they do not trust nutritionists who are affiliated with large feed companies</td>
</tr>
</table>
